# The Identification of Children at Fever Hospitals

**Published:** 1898-01-08

**Authors:** 


### THE IDENTIFICATION OF CHILDREN AT FEVER HOSPITALS.

A very distressing occurrence has taken place at the North-Western Fever Hospital. Two children appear to have been admitted on the same day, and by some blunder an interchange of names was made. One of the children died, and thus it happened that while the parents of the recovering patient were informed that their child was seriously ill—while they visited it, and ultimately buried it—the parents of the child that died were repeatedly assured that he was going on well, and only discovered the error when, on arriving at the hospital to take him home, they discovered that he was not their child, but one belonging to some one else. Then the sad truth came out. The extraordinary part of the story is that three relatives of the child that lived—the father, the grandfather, and the brother-in-law—when sent for, came and sat by the dying child, never found out the mistake, and ultimately buried him as their own. This is not the first time that mistakes of this kind have occurred, although not, we believe, involving such distressing results. In response to our inquiries as to the means taken to prevent such an event, Mr. Duncombe Mann, the clerk to the Asylums Board, has forwarded to us a copy of the Instructions to Nurses employed on Ambulance Duty, from which it would appear that, if the nurses follow accurately the instructions given, it should be impossible for such an accident to happen. At the same time, they show how easy it would be for children to get changed unless the rules are systematically obeyed. The nurses are instructed, unless the patients or friends wish otherwise, to leave all outer clothing at the house and to wrap the patient carefully in the clothing and blankets taken from the hospital, none of its own clothes being worn except the vest and chemise, or shirt. It seems pretty clear then that if two children, so deprived of means of identification, were to arrive together, an interchange might easily occur were not some special means taken to prevent such an accident. But among the instructions is the following: "15. Before removing any child of 7 years or under from its home to the ambulance, to tie round its neck a label with the child's name, age, and date of removal written on it; and to hand the child over to the nurse in charge with the label tied round its neck." According to this, the label ought to be put on before the child leaves the house, and if the label is kept on these regulations ought to be sufficient. The matter would seem to be one of discipline. On paper such an accident seems impossible, but if discipline is slack all things are possible.

